# Independent lung ventilation in a newborn with asymmetric acute lung injury due to respiratory syncytial virus: a case report

**DOI:** 10.1186/1752-1947-2-212

**Published:** 2008-06-19

**Authors:** Matteo Di Nardo, Daniela Perrotta, Francesca Stoppa, Corrado Cecchetti, Marco Marano, Nicola Pirozzi

**Affiliations:** 1 Ospedale Pediatrico Bambino Gesu'-IRCS, Rome, Italy

## Abstract

**Introduction:**

Independent lung ventilation is a form of protective ventilation strategy used in adult asymmetric acute lung injury, where the application of conventional mechanical ventilation can produce ventilator-induced lung injury and ventilation-perfusion mismatch. Only a few experiences have been published on the use of independent lung ventilation in newborn patients.

**Case presentation:**

We present a case of independent lung ventilation in a 16-day-old infant of 3.5 kg body weight who had an asymmetric lung injury due to respiratory syncytial virus bronchiolitis. We used independent lung ventilation applying conventional protective pressure controlled ventilation to the less-compromised lung, with a respiratory frequency proportional to the age of the patient, and a pressure controlled high-frequency ventilation to the atelectatic lung. This was done because a single tube conventional ventilation protective strategy would have exposed the less-compromised lung to a high mean airways pressure. The target of independent lung ventilation is to provide adequate gas exchange at a safe mean airways pressure level and to expand the atelectatic lung. Independent lung ventilation was accomplished for 24 hours. Daily chest radiograph and gas exchange were used to evaluate the efficacy of independent lung ventilation. Extubation was performed after 48 hours of conventional single-tube mechanical ventilation following independent lung ventilation.

**Conclusion:**

This case report demonstrates the feasibility of independent lung ventilation with two separate tubes in neonates as a treatment of an asymmetric acute lung injury.

## Introduction

Independent lung ventilation (ILV) is generally used in adult asymmetric lung injury because the application of conventional pressure controlled ventilation (PCV) with positive end-expiratory pressure to both lungs can overdistend the less-compromised lung, diverting pulmonary blood flow to the injured lung areas, worsening ventilation perfusion mismatch [1]. Pressure controlled high-frequency ventilation (HFPPV) is a form of protective ventilation that uses high respiratory rates (150 breaths per minute) with small tidal volume, allowing low mean airway pressure to theoretically reduce ventilation induced lung injury (VILI).

We report the case of a 16-day-old infant, ventilated with two different tubes and different ventilation strategies to treat a severe respiratory syncytial virus bronchiolitis with asymmetric lung injury, which had been unresponsive to conventional pharmacological therapy and single-tube mechanical ventilation.

## Case presentation

**Figure 1:**
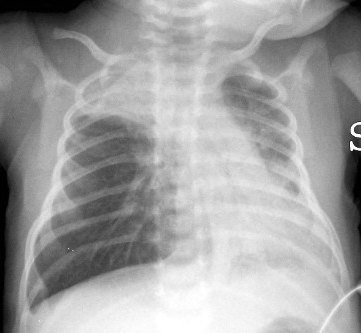
Chest X-ray on admission to the emergency room.

A male neonate of 16 days, 3.5 kg body weight, was brought to our emergency department with severe respiratory distress. One day before admission, he had suddenly developed progressive dyspnea, high respiratory rate and wheezing. On admission oxygen saturation (SpO_2_; determined by pulsoximetry) in breathing room air was 65%. There was no fever. His hemodynamic conditions were stable and arterial blood gas analysis (ABG) revealed the following values: pH, 7.14; PaO_2_/FiO_2_, 149; PaCO_2_, 75 mmHg; HCO_3_-, 21 mEq/liter; Base Excess (BE), -8. A chest radiograph demonstrated lung hyperinflation with a flattened diaphragm and bilateral atelectasis in the right apical and left basal regions (Figure 1). Diagnosis of respiratory syncytial virus (RSV) bronchiolitis was quickly made through the detection of RSV antigen on pharyngeal secretion using an enzyme-linked immuno-absorbent assay (ELISA) method [2].

**Figure 2:**
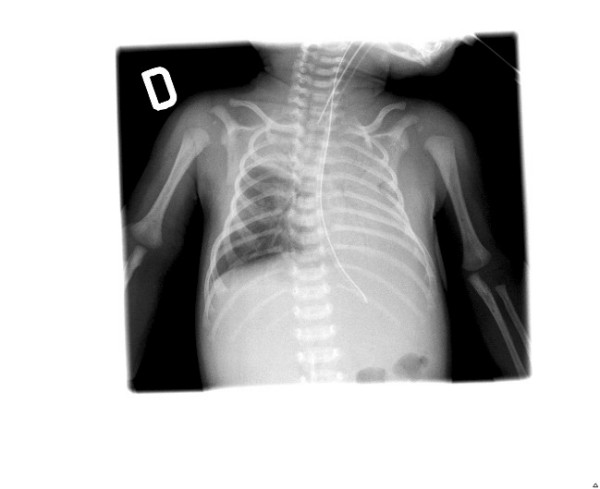
Chest X-ray after 48 hours of PCV.

Initially manual ventilation and then supplemental humidified oxygen, inhaled bronchodilators and intravenous corticosteroids led to a mild clinical improvement in the patient's condition, but this treatment did not sufficiently increase gas exchange or lead to a sufficient improvement on the chest radiograph. Therefore, after 72 hours and worsening of clinical features, including frequent apnoeic episodes, we decided to start mechanical ventilation with oral intubation, sedation and muscular relaxation. Twenty-four hours of PCV resulted in the following conditions: peak inspiratory pressure, 25 cmH_2_O; positive end expiratory pressure (PEEP), 3 cmH_2_O (intrinsic PEEP (PEEPi), 5 cmH_2_O); respiratory rate, 45 breaths per minute; I:E, 1:1.5; FiO_2_, 80% with an achieved tidal volume of approximately 21 to 25 ml (6 to 7 ml/kg) and a mean airways pressure of 14 to 15 cmH_2_O. The airways pressure sensor line was located between the extremity of the endotracheal tube and the Y-piece of the external circuit of the ventilator. ABG values did not improve (pH, 7.19; PaCO_2_, 70 mmHg; PaO_2_/FiO_2_, 68; HCO_3_-, 26 mEq/liter; BE, -5) and the chest radiograph showed asymmetric changes with severe left atelectasis and right lung hyperinflation (Figure 2).

As well as PCV, the patient was changed to the right decubitus position and had three left bronchoalveolar lavages with 1 ml/kg of saline solution 0.9% to eliminate mucus plugs. No recruiting maneuvers were attempted, because we considered them unsafe for bronchiolitis management. The mean airways pressure increased to 16 cmH_2_O with the increase of total PEEP accorded to a PEEPi of 6 to 7 cmH_2_O. Transthoracic echocardiography revealed a leftward septal shift as a sign of right ventricle pressure overload, and so we decided to start ILV [3] to expand the collapsed left lung and to reduce the risk of VILI in the less-compromised right lung. We used two 2 mm tubes to attempt ILV. We positioned one tube in the trachea to ventilate the left lung and the other tube at the beginning of the right main bronchus. Positioning was controlled with chest radiographic guidance using fluoroscopy while inserting the tubes. Both lungs were ventilated asynchronously with two Siemens Ventilators 300/300A (Siemens Sweden) in PCV.

**Figure 3:**
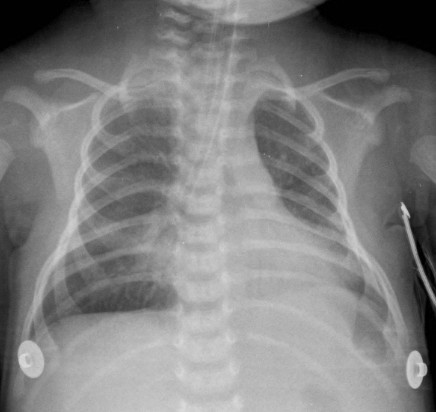
Chest X-ray after 24 hours of ILV.

The less-compromised right lung was ventilated in PCV with: peak inspiratory pressure, 8 cmH_2_O; PEEP, 3 cmH_2_O; respiratory rate, 50 breaths per minute; I:E, 1:1.5; FiO_2_, 40% to reach a tidal volume of approximately 10 ml (3 ml/kg); and a mean airways pressure of 4 cmH_2_O. The more-compromised left lung was treated with HFPPV in PCV with a peak pressure of 15 cmH_2_O, a high respiratory rate frequency (150 breaths per minute), a PEEP of 4 cmH_2_O, I:E of 1:1, FiO_2 _of 40%, reaching a tidal volume of almost 10 ml (3 ml/kg) and a mean airways pressure of 8 cmH_2_O. Permissive hypercapnia of 70 mmHg was tolerated in the first 24 hours. After a period of 24 hours there was a progressive re-opening of the collapsed left lung (Figure 3) with improvement of ABG (pH, 7.36; PaO_2/_FiO_2_, 277; PaCO_3_, 45 mmHg; HCO_3_-, 25 mEq/liter; BE, -2).

On the basis of these results ILV was discontinued and the patient was ventilated with conventional PCV (peak pressure, 16 cmH_2_O; PEEP, 4 cmH_2_O; respiratory rate, 50 breaths per minute; I:E, 1:1.5; FiO_2_, 30%) for another 2 days before extubation. Blood gas before extubation was: pH, 7.38; PaO_2_/FiO_2_, 356; PaCO_2_, 45 mmHg; BE, 5; HCO_3_-, 24 mEq/liter). The patient was discharged (pH, 7.48; PaO_2_/FiO_2_, 450; PaCO_2_, 35 mmHg; HCO_3_-, 22; BE, 4) from the pediatric intensive care unit to the pneumology ward 9 days after admission.

## Conclusion

Mean airways pressure limitation is now a largely accepted strategy in adult acute lung injury; however, there is debate about the exact level of mean airways pressure that can be used safely [4]. Tidal volume and PEEP also play an important role in VILI management. In our institution we use HFPPV with a pressure-controlled mode, paying attention to the tidal volumes reached indirectly (4 to 6 ml/kg), to the mean airways pressure (chosen according to an echocardiography report) and to the total PEEP (70% of PEEPi).

To the best of the authors' knowledge, at present there are no guidelines on the best lung protective strategy and the safe mean airways pressure that can reduce or avoid VILI in neonates. For this reason in our institution we measure, using echocardiography, pulmonary systolic arterial pressure daily in all mechanically ventilated newborns as an indirect indicator of alveolar overdistension [5]. We have realized that the application of a high mean airways pressure (> 15 to 16 cmH_2_O) to a newborn's airways increases the pulmonary systolic arterial pressure. This adverse effect could be considered to be the result of airway distension, since pulmonary capillaries, as intra-alveolar vessels, are collapsed by alveolar pressure. This can lead to a progressive right ventricular dysfunction which may affect the patient's outcome. For this reason in this patient, after 48 hours of conventional mechanical ventilation with no improvement in gas exchange or chest X-ray and with leftward septal shift on transthoracic echocardiography, we decided to start ILV.

Few reports on the use of ILV are related to the management of severe air leak or asymmetric acute lung injury in pediatric patients [6]. To the best of the authors' knowledge, this is the first report of a case of ILV where two separate tubes were used for the treatment of severe bronchiolitis in a neonate.

ILV is rarely used in pediatric patients [7,8], but when it is indicated we use a pediatric Marraro's double lumen tube. In infants older than 1 year, this double lumen tube fits well, allowing rapid selective intubation. In newborns of different weight, intubation with a Marraro's tube is not able to be easily performed, because of the frequent anatomic variability of the airways. Experiences with a Marraro's double lumen tube in newborns are limited in the published literature, so in order to avoid possible tracheal-bronchial lesions, we performed double intubation with two small tubes. This technique in our experience is easy and rapid in neonates and allows ILV with just the assistance of radiographic control while inserting the tubes to evaluate their position. Two tubes can be unstable with the possibility of displacement when inserted via the mouth, especially during nursing. This rarely happens when intubations are performed via the nasal passages. Attention should be paid to the risk of airway harm caused by prolonged ILV.

Some institutions have used ILV in pediatric patients with conventional ventilation and high-frequency oscillatory ventilation (HFOV) as a strategy to minimize barotrauma and alveolar overdistension [9]. We believe that in centers with no HFOV machine it is possible to keep the lung open with low mean airways pressure by using HFPPV. This protective ventilation strategy opens lung units with a mean airways pressure chosen directly by the clinician, who sets the peak inspiratory pressure on the ventilator. The small tidal volumes achieved avoid the problem of hemodynamic instability due to asynchronous ventilation.

This case report demonstrates the feasibility and efficacy of ILV with two separate tubes to treat asymmetric acute lung injury in newborns.

## Abbreviations

ABG: arterial blood gas; BE: base excess; HFPPV: high frequency positive pressure ventilation; iPEEP: intrinsic positive end expiratory pressure; ILV: independent lung ventilation; PCV: pressure controlled ventilation; PEEP:positive end expiratory pressure; RSV: respiratory syncytial virus; VILI: ventilator induced lung injury.

## Competing interests

The authors declare that they have no competing interests.

## Consent

Written consent was obtained from the legal guardian of the patient for publication of this case report and any accompanying images. A copy of the written consent is available for review by the Editor-in-Chief of this journal.

## Authors' contributions

MD wrote this article and carried out the management of the patient, DP and FS performed ILV and carried out the management of the patient, CC, MM and NP carried out the management of the patient and helped to draft the manuscript.
